# Tear-drop technique in iliac screw placement: a technical analysis

**DOI:** 10.1007/s00701-021-04788-1

**Published:** 2021-03-05

**Authors:** Stephan Nowak, Jonas Müller, Martin E. Weidemeier, Henry W. S. Schroeder, Jan-Uwe Müller

**Affiliations:** Universitätsmedizin Greifswald, Klinik und Poliklinik für Neurochirurgie, Sauerbruchstraße, 17475 Greifswald, Germany

**Keywords:** Iliac screw, Tear-drop, Spine, Surgical technique, Surgery, X-ray, Screw placement, Spinal surgery

## Abstract

**Background:**

Instrumentation of the lumbosacral region is one of the more challenging regions due to the complex anatomical structures and biomechanical forces. Screw insertion can be done both navigated and based on X-ray verification. In this study, we demonstrate a fast and reliable open, low exposure X-ray-guided technique of iliac screw placement.

**Methods:**

Between October 2016 and August 2019, 48 patients underwent sacropelvic fixation in tear-drop technique. Screw insertion was performed in open technique by using an X-ray converter angulated 25-30° in coronal and sagittal view. The anatomical insertion point was the posterior superior iliac spine. Verification of correct screw placement was done by intraoperative 3D scan.

**Results:**

In total, 95 iliac screws were placed in tear-drop technique with a correct placement in 98.1%.

**Conclusions:**

The tear-drop technique showed a proper screw position in the intraoperative 3D scan and therefore may be considered an alternative technique to the navigated screw placement.

## Introduction

Instrumentation of the lumbosacral region is one of the more challenging regions due to the complex anatomical structures and biomechanical forces [5, 14]. Especially multilevel fusions including the sacral area for adult deformity, lumbosacral instability, or stenosis are associated with poor clinical outcome. Often, the rates of major complications, pseudarthrosis (up to 33%), and implant failure are high [4]. To reduce the rate of the aforementioned complications, multiple techniques to support the S1 pedicle screws were developed. It has been demonstrated that additional iliac fixation enhances the fusion rate of the lumbosacral junction [9, 11]. Numerous techniques have been developed for iliac fixation [15]. Especially S2 ala-iliac and iliac screws, both techniques lead to biomechanical good results [1, 12]. Due to no prominence of the screwhead in thin patients and the possibility to directly connect to the fixation system without offset connectors, the S2 ala-iliac screws are preferred. Next to open surgical techniques for iliac fixation with anatomic guidance, also navigated or robotic-supported placements have been described [8, 14]. Already good to excellent accuracy can be achieved with these technically assisted methods. Under robotic guidance, an accuracy rate of 95.7% can already be achieved [10]. Only free-hand technique followed by navigated screw placement can achieve better accuracy with 100% vs. 98% respectively [8]. These techniques, if applied correctly, have the advantage to give the surgeon an extra reference for safety at the cost of prolonged operative time and radiation exposure relative to conventional fluoroscopy-based methods [2, 7, 13]. That is why we adapted a fast and simple radiological controlled open placement technique for iliac screw fixation at our neurosurgical spine center.

## Materials and methods

Between October 2016 and August 2019, 48 patients underwent sacropelvic fixation in tear-drop technique. Between 2016 and 2019, all patients who received iliac screw placement were included. Only in one case, iliac screw placement was achieved in navigated technique (due to neoplastic sacrum deformity). This case was excluded. To indicate surgery, we applied generally accepted standards in every specific case. The preoperative diagnostic procedure included a thorough anamnesis and clinical examination. The preoperative imaging included an MRI and CT of the lumbar spine. Intraoperative screw position was verified with a 3D X-ray scan with c-arm. Follow-up X-ray was performed before discharge and 3 months after surgery. The data was collected prospectively. The chart review was done retrospectively. The statistical analysis was done by using Excel and SPSS for Windows.

## Surgical technique

All surgical procedures were performed by our senior spine surgeon and senior author (J.-U. Müller). Our spinal instrumentation system and pedicle screws used in all cases of this study are from Signus Medizintechnik (Signus GmbH, Alzenau, Germany).

**Fig. 1:**
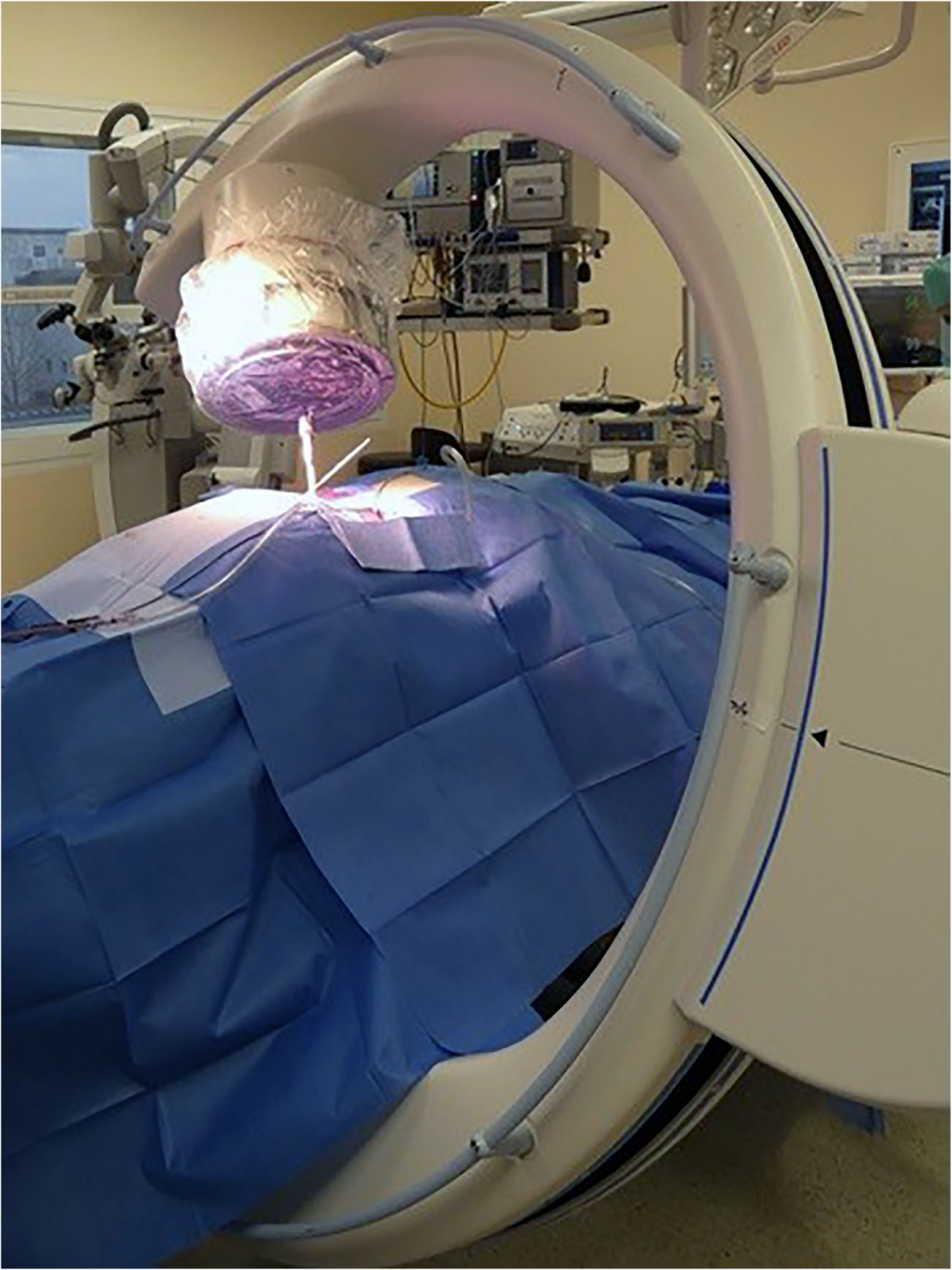
Correct X-ray alignment in 25° sagittal and coronal plane with inserted awl

**Fig. 2:**
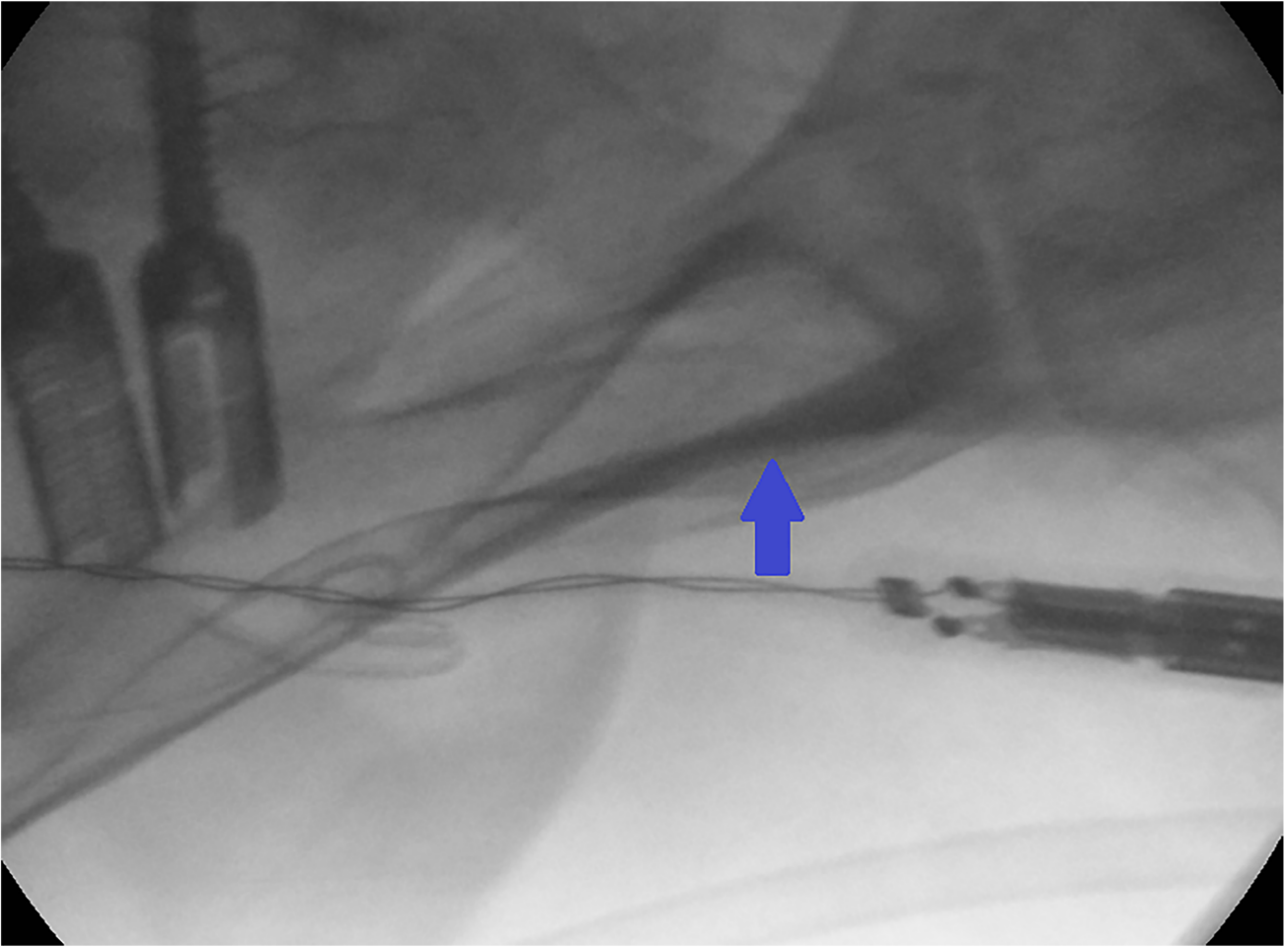
Cortical layers of the iliac bone from the “tear-drop sign” (blue arrow)

**Fig. 3:**
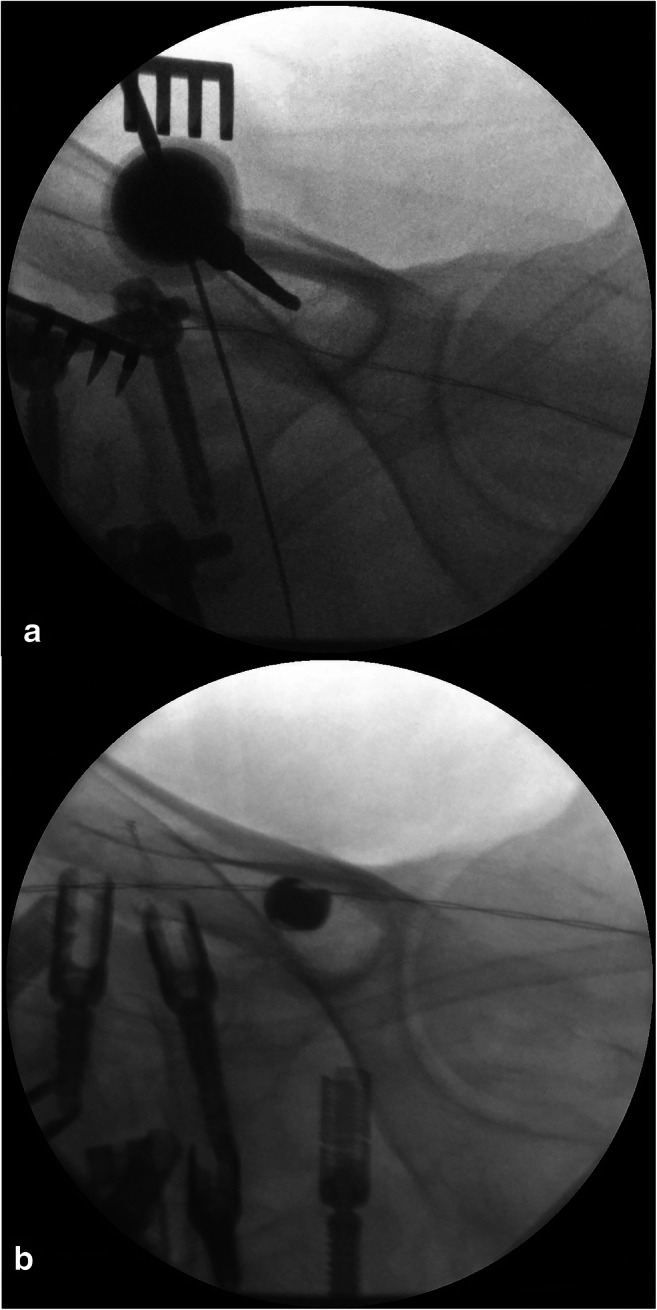
Correct placement of the iliac screw in “tear-drop” technique without wall breach. **a** Correct insertion of the awl inside the tear-drop sign. **b** Correct placement of the iliac screw

Surgery is performed in general anesthesia with the patient being in prone position. Spinal exposure is obtained via a standard midline incision from the lumbar spine to the sacrum. The distal extent of the incision should be lower than the line between the posterior superior iliac spine (PSIS). The traditional anatomical landmark for iliac screw fixation is the PSIS [9]. The bony PSIS will be exposed bluntly. The X-ray converter will be angled 25-30° in coronal and sagittal plane with a prone position of the patient, unlike the iliac oblique view where the patient is rotated 45° by wedge elevation, leading to a completely different pelvic image. Figure 1 shows the correct intraoperative X-ray alignment. To confirm proper X-ray view, a “tear-drop sign,” as depicted in Fig. 2, must be visible. The outer edges of the “tear-drop sign” will be formed by the cortical layers of the iliac bone. The tear-drop sign will lead the direction of the screw insertion. The screw has to be placed directly in the middle of the tear-drop sign. Ideally there is a direct look on view of the screw inside the tear-drop as depicted in Fig. 3. The burr channel will be prepared medial to the PSIS via a bone awl followed by a proper check using a probe. Then, a guide wire will be inserted under guidance of the tear-drop sign. The medial PSIS will be opened with a chisel for the screw head which should later sink into the burr hole. This step is important as otherwise the screw head can be prominent under the skin, even more so in skinny patients. Commonly an 8.5 × 80 mm screw will be inserted. We use preoperative imaging and intraoperative probe to determine the best suitable screw length and diameter. The screw should not breach through the tear-drop sign in the X-ray overview. After screw placement, a 3D X-ray will be performed to rule out sciatic notch or acetabular breach. The iliac screws will be connected to the screw rod via an offset connector. We are convinced that a direct connection with the lumbosacral fixation system would bring too much force on the distal rod thus leading frequently to rod fracture and pseudarthrosis. In the postoperative period, the patient will be hospitalized for 1 week. Before discharge, a common X-ray will be performed.

## Results

Between October 2016 and August 2019, we included 48 patients. A total of 27 of them were female and 21 male. The mean age was 64 years (27-81 years). In all cases, bilateral iliac screw positioning was performed. In total, 95 iliac screws were placed in tear-drop technique. Only in one side (1.04%), navigated placement was performed due to tumor which deformed the iliac anatomy. The mean follow-up was 9.8 months, ranging from 7 days to 27 months. Spinal instrumentation was performed due to degenerative spinal scoliosis in 22 cases (45.8%). In an equal number (22 cases; 45.8%), a revision surgery after previous spinal instrumentation needed spinopelvic fusion. Of that group, there were 7 cases with pseudarthrosis of L5/S1 (14.6% from the whole cohort). All cases received a multilevel spinal instrumentation. Pseudarthrosis of the sacroiliac joint after multilevel spinal instrumentation was seen in 3 cases (6.2% of the whole cohort). Spinal trauma involving the sacrum and pelvic tumor infiltration contributed 2 cases each (4.2%). On average, 8 X-ray images were needed to adjust the tear-drop sign, make the pre-drilling, and place the screw on one side. The radiation exposure depends on the X-ray converter used and the technical settings applied by the surgeon. On average, our X-ray converter takes 83kV and 9.1mA. In all cases but one, the 3D X-ray scan after tear-drop screw placement confirmed a correct position of the screw (98.9%). In one case, there was a lateral breach of the screw head. Prior to the 3D X-ray scan, no redirection of a screw was needed. In the 3-month follow-up, there were no complications regarding the iliac screws. In the overall follow-up time, there were no complications involving the iliac screw, up until today. Other surgical complications included postoperative infection in 4 cases (8.3%), non-fusion of the upper and mid-level in 4 cases (8.3%), and fractured pedicle screw on level L2 in one case (2.1%).

## Discussion

The addition of iliac fixation should be considered in multilevel spinal fusion including the lumbosacral crossover in adult deformity and instability to reduce the risk of lumbosacral pseudarthrosis [9, 11]. In our institution, we use the tear-drop technique for iliac screw placement in every case of normal pelvic anatomy. We only switch to navigated screw placement in case of trauma with changed pelvic anatomy, congenital spinal and pelvic derangement, or instability due to tumor infiltration with pelvic involvement.

The radiographically guided tear-drop technique combines the advantages of both free-hand and navigated placement techniques. The exposure level is minimal, as we use the existing surgical corridor for spinopelvic fusion. Only a minimal exposure of the PSIS will be performed for visualization and preparation of the entry point. Additionally, the radiological exposure level will be low. The tear-drop sign must be adjusted on the X-ray monitor and the screw placed under radiological guidance. We recommend, if available, a 3D scan directly after screw placement in open as well as in navigated placement to ensure a correct screw position without breach.

The technique necessitates at least a c-arm X-ray apparatus and a carbon surgical table to ensure visualization. Also, it is important to align the tear-drop sign in the central X-ray beam as otherwise a visual aberration can lead to wrong screw position leading to screw breach. A recent meta-analysis comparing iliac screw placement with S2 ala-iliac screw placement detected mechanical complications in up to 27.9% of the cases vs. 14.2% respectively [3]. Our rate of wound infections is 8.6% and for being related to a complex spinal procedure, we consider this an acceptable range [16]. It is important to note though that the overall infection rate depends strongly on other factors of the performed procedure (i.e., total number of screws being placed, level of fusion, trauma) and is therefore not a viable indicator for pure iliac screw safety.

When comparing to other free-hand techniques, as for example proposed by Fridley et al., we achieve comparable results [6]. In their technique, no X-ray guidance is used. However, we would like to note that a radiological reference often will be needed due to anatomical variations, listhesis, trauma, or tumor.

## Conclusion

The tear-drop technique for iliac screw placement in spinal surgery is a reliable and safe method only utilizing standard X-ray imaging and minimal exposure of the surgical field.
